# 2-Benzyl-*myo*-inositol monohydrate

**DOI:** 10.1107/S1600536809041750

**Published:** 2009-10-17

**Authors:** Richard F. G. Fröhlich, Graeme J. Gainsford, Gavin F. Painter

**Affiliations:** a Industrial Research Limited, PO Box 31-310, Lower Hutt, New Zealand

## Abstract

The title structure, C_13_H_18_O_6_·H_2_O, contains two independent 2-benzyl-*myo*-inositol and water mol­ecules. In the crystal, the mol­ecules are strongly hydrogen bonded into an infinite two dimensional network utilizing all OH protons.

## Related literature

For puckering parameters, see: Cremer & Pople (1975 ▶). For related structures, see: Khan *et al.* (2007 ▶); Simperler *et al.* (2006 ▶); Gibson *et al.* (2009 ▶).
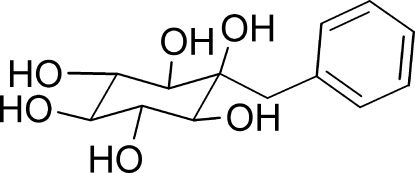


## Experimental

### 

#### Crystal data

C_13_H_18_O_6_·H_2_O
*M* *_r_* = 288.29
Monoclinic, 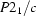

*a* = 7.4616 (3) Å
*b* = 33.7688 (13) Å
*c* = 10.4528 (4) Å
β = 90.616 (2)°
*V* = 2633.63 (18) Å^3^
*Z* = 8
Mo *K*α radiation
μ = 0.12 mm^−1^
*T* = 121 K
0.75 × 0.72 × 0.31 mm

#### Data collection

Bruker APEXII CCD diffractometer
Absorption correction: multi-scan (Blessing, 1995 ▶) *T*_min_ = 0.657, *T*_max_ = 0.746
67666 measured reflections
8428 independent reflections
7368 reflections with *I* > 2σ(*I*)
*R*_int_ = 0.031

#### Refinement

*R*[*F* ^2^ > 2σ(*F* ^2^)] = 0.041
*wR*(*F* ^2^) = 0.110
*S* = 1.08
8428 reflections
385 parameters
H atoms treated by a mixture of independent and constrained refinement
Δρ_max_ = 0.54 e Å^−3^
Δρ_min_ = −0.26 e Å^−3^

### 

Data collection: *APEX2* (Bruker, 2005 ▶); cell refinement: *SAINT* (Bruker, 2005 ▶); data reduction: *SAINT* and *SADABS* (Bruker, 2005 ▶); program(s) used to solve structure: *SHELXS97* (Sheldrick, 2008 ▶); program(s) used to refine structure: *SHELXL97* (Sheldrick, 2008 ▶); molecular graphics: *ORTEP-3* (Farrugia, 1997 ▶), *Mercury* (Macrae, 2006 ▶) and *PLATON* (Spek, 2009 ▶); software used to prepare material for publication: *SHELXL97* and *PLATON*.

## Supplementary Material

Crystal structure: contains datablocks global, I. DOI: 10.1107/S1600536809041750/ez2193sup1.cif

Structure factors: contains datablocks I. DOI: 10.1107/S1600536809041750/ez2193Isup2.hkl

Additional supplementary materials:  crystallographic information; 3D view; checkCIF report

## Figures and Tables

**Table 1:** Hydrogen-bond geometry (Å, °)

*D*—H⋯*A*	*D*—H	H⋯*A*	*D*⋯*A*	*D*—H⋯*A*
O1′—H1′*O*⋯O1*W*^i^	0.84	1.87	2.7052 (11)	175
O1—H1*O*⋯O6′^i^	0.84	1.94	2.7768 (10)	171
O2′—H2′*O*⋯O2*W*^ii^	0.84	1.90	2.7267 (12)	166
O2—H2*O*⋯O3′^iii^	0.84	2.04	2.7755 (10)	145
O3′—H3′*O*⋯O4^iv^	0.84	2.01	2.8420 (10)	174
O3—H3*O*⋯O2′^iv^	0.84	2.09	2.9290 (11)	172
O4′—H4′*O*⋯O6^v^	0.84	1.91	2.7389 (10)	168
O4—H4*O*⋯O5′	0.84	1.87	2.6858 (10)	165
O5′—H5′*O*⋯O4′^iii^	0.84	1.86	2.6943 (11)	175
O5—H5*O*⋯O4^ii^	0.84	2.57	3.3617 (11)	157
O6′—H6′*O*⋯O5	0.84	2.13	2.8523 (11)	144
O6—H6*O*⋯O1′^ii^	0.84	2.05	2.8831 (10)	173
O1*W*—H1*WB*⋯O2	0.85 (2)	1.96 (2)	2.8108 (12)	174.2 (19)
O2*W*—H2*WA*⋯O1	0.85 (2)	1.91 (2)	2.7521 (11)	173.3 (18)
O1*W*—H1*WA*⋯*Cg*1^vi^	0.82 (2)	2.59 (2)	3.2647 (11)	140.9 (19)
O2*W*—H2*WB*⋯*Cg*2^i^	0.83 (2)	2.59 (2)	3.3335 (11)	149.9 (19)

Symmetry codes: (i) 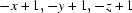; (ii) 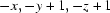; (iii) 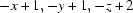; (iv) 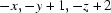; (v) 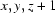; (vi) 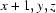.

**Fig. 1.**
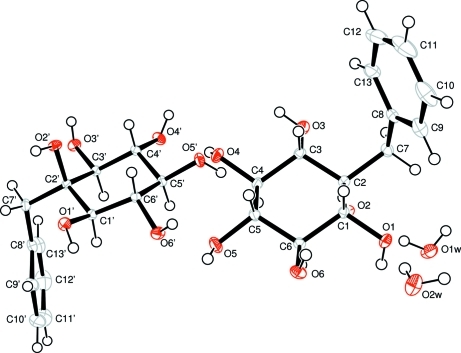
Asymmetric unit contents of (I) (Farrugia, 1997).

**Fig. 2.**
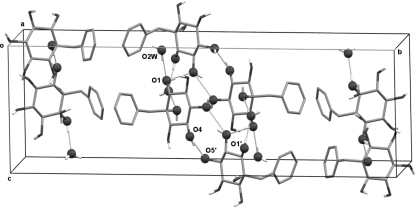
Packing diagram (Macrae et al., 2006) of (I) viewed down the a axis with some atom labels. Some hydrogen bonds are shown as dashed lines (see text); H atoms not involved in hydrogen bonds are omitted for clarity.

## supplementary crystallographic information

### Comment

The enzyme myo-inositol oxygenase (MIOX) is over expressed in the kidney tissue of patients suffering from type II diabetes mellitus and defects in the metabolism of inositol sugars are also strongly associated with diabetes. Because MIOX is responsible for the first committed step in the catabolism of inositols, we were interested in developing chemical inhibitors of this enzyme. 2-Benzyl-*myo*-inositol is a candidate substrate-like (enzyme) inhibitor: specifically, inclusion of the equatorial benzyl group was designed to take advantage of a hydrophobic binding pocket identified adjacent to the MIOX active site.

The asymmetric unit of the title compound (I) contains two independent 2-benzyl-*myo*-inositol molecules and two waters of crystallization. The two molecules (Fig. 1) are essentially identical with chirality designations for carbons 1,2,4, & 6 of of *R*,*S*,*R*, & S and with the non-hydrogen atoms having a r.m.s. bond fit of 0.0043Å & the bond angles an r.m.s. fit of 1.93° (Spek, 2009). There is a slight twist between the two rings; torsion angles φ are C2—C7—C8—C13 - 88.67 (13),-92.34 (12)° for unprimed & primed molecules respectively. The inositol rings are in chair conformations with Cremer & Pople (1975) parameters Q, θ, φ of 0.5601 (10),0.5689 (10) Å, 176.85 (10),172.98 (10)° and 275.6 (19),333.1 (8)° respectively compared with 0.576 (2) Å, 177.9 (2) and 245 (7)° for a "parent" myo-inositol (Khan *et al.*, 2007).

An extensive set of 14 hydrogen bonds involves nearly all available donors and acceptors (Table 1); all the hydrogen donors were located from difference maps, but with only the water hydrogen atomic positions refined with restrained thermal parameters. The two remaining water hydrogen atoms (H1WB & H2WB) are involved in O–H···π interactions with the phenyl rings (Table 1: *Cg*1,*Cg*2 are the centres of phenyl rings C8—C13 & C8'-C13' respectively). Overall, the molecules are strongly hydrogen bonded into an infinite two dimensional network with the benzyl groups making van der Waal contacts between the layers (Fig. 2). One antiparallel "double chain" link is observed in the complex system reflecting aspects of *myo*-inositol packing (Simperler *et al.*, 2006).

### Experimental

2-Benzyl-1,3,4,5,6-penta-*O*-benzyl-*myo*-inositol (78 mg, 0.11 mmol) (Gibson *et al.*, 2009) was dissolved in THF (5 ml) and palladium hydroxide (20% on carbon, wet, 65 mg) was added; the air was replaced with hydrogen (gasbag, 1 atm) and the mixture was stirred at RT for 1 h. The flask was aerated and left overnight. Next morning the solution was filtered through a pad of filter-aid, rinsed with MeOH and preabsorbed on silica (~0.5 g) and purified by chromatography (9 g silica, CHCl_3_/MeOH = 3:1 *v*/*v*) which gave the product as a white solid. This was recrystallized from MeOH (25 mg, 85%). ^1^H-NMR (500 MHz, methanol-d4) δ 7.47–7.37 (m, 2H), 7.28–7.22 (m, 2H), 7.21–7.15 (m, 1H), 3.56 (t, *J* = 9.4 Hz, 2H), 3.05 (s, 2H), 2.99 (d, *J* = 9.4 Hz, 2H), 2.84 (t, *J* = 9.4 Hz, 1H); ^13^C-NMR (126 MHz, methanol-d4) δ 138.4, 131.8, 129.1, 127.4, 77.9, 75.8, 75.4, 72.4, 40.8; HRMS (*M*+Na)+ C_13_H_18_O_6_Na: calcd 293.1001; found 293.1004; micro anal. for C_13_H_18_O_6_.H_2_O: C (calcd. 54.16%) 54.64; H (6.99%) 6.97.

### Refinement

All H atoms on the inositol molecules were constrained to their expected geometries [C–H 0.95, 0.99 & 1.00; O–H 0.84 Å] with *U*_iso_ = 1.2 *U*_eq_ of the parent atom. All water H atoms were located on difference Fourier maps and refined with *U*_iso_ = 1.5 *U*_eq_ (O).

### Crystal data

**Table:**

C_13_H_18_O_6_·H_2_O	*F*(000) = 1232
*M**_r_* = 288.29	*D*_x_ = 1.454 Mg m^−^^3^
Monoclinic, *P*2_1_/*c*	Mo *K*α radiation, λ = 0.71073 Å
Hall symbol: -P 2ybc	Cell parameters from 9735 reflections
*a* = 7.4616 (3) Å	θ = 2.4–31.2°
*b* = 33.7688 (13) Å	µ = 0.12 mm^−^^1^
*c* = 10.4528 (4) Å	*T* = 121 K
β = 90.616 (2)°	Block, colourless
*V* = 2633.63 (18) Å^3^	0.75 × 0.72 × 0.31 mm
*Z* = 8	

### Data collection

**Table:**

Bruker APEXII CCD diffractometer	8428 independent reflections
Radiation source: fine-focus sealed tube	7368 reflections with *I* > 2σ(*I*)
graphite	*R*_int_ = 0.031
Detector resolution: 8.333 pixels mm^-1^	θ_max_ = 31.3°, θ_min_ = 2.0°
φ and ω scans	*h* = −10→10
Absorption correction: multi-scan (Blessing, 1995)	*k* = −48→48
*T*_min_ = 0.657, *T*_max_ = 0.746	*l* = −15→14
67666 measured reflections	

### Refinement

**Table:**

Refinement on *F*^2^	Primary atom site location: structure-invariant direct methods
Least-squares matrix: full	Secondary atom site location: difference Fourier map
*R*[*F*^2^ > 2σ(*F*^2^)] = 0.041	Hydrogen site location: inferred from neighbouring sites
*wR*(*F*^2^) = 0.110	H atoms treated by a mixture of independent and constrained refinement
*S* = 1.08	*w* = 1/[σ^2^(*F*_o_^2^) + (0.0524*P*)^2^ + 1.1394*P*] where *P* = (*F*_o_^2^ + 2*F*_c_^2^)/3
8428 reflections	(Δ/σ)_max_ = 0.001
385 parameters	Δρ_max_ = 0.54 e Å^−^^3^
0 restraints	Δρ_min_ = −0.26 e Å^−^^3^

### Special details

**Table:**

Geometry. All e.s.d.'s (except the e.s.d. in the dihedral angle between two l.s. planes) are estimated using the full covariance matrix. The cell e.s.d.'s are taken into account individually in the estimation of e.s.d.'s in distances, angles and torsion angles; correlations between e.s.d.'s in cell parameters are only used when they are defined by crystal symmetry. An approximate (isotropic) treatment of cell e.s.d.'s is used for estimating e.s.d.'s involving l.s. planes.
Refinement. Refinement of *F*^2^ against ALL reflections. The weighted *R*-factor *wR* and goodness of fit *S* are based on *F*^2^, conventional *R*-factors *R* are based on *F*, with *F* set to zero for negative *F*^2^. The threshold expression of *F*^2^ > σ(*F*^2^) is used only for calculating *R*-factors(gt) *etc*. and is not relevant to the choice of reflections for refinement. *R*-factors based on *F*^2^ are statistically about twice as large as those based on *F*, and *R*- factors based on ALL data will be even larger.

### Fractional atomic coordinates and isotropic or equivalent isotropic displacement parameters (Å^2^)

**Table:**

	*x*	*y*	*z*	*U*_iso_*/*U*_eq_	
O1	0.50902 (10)	0.38866 (2)	0.32399 (7)	0.01471 (14)	
H1O	0.5917	0.4055	0.3203	0.018*	
O2	0.60347 (10)	0.41015 (2)	0.57055 (7)	0.01483 (14)	
H2O	0.6332	0.4083	0.6481	0.018*	
O3	0.35266 (11)	0.39608 (3)	0.76876 (7)	0.01909 (16)	
H3O	0.2746	0.4030	0.8216	0.023*	
O4	0.11845 (10)	0.46202 (2)	0.71154 (7)	0.01597 (14)	
H4O	0.1612	0.4703	0.7812	0.019*	
O5	0.19999 (12)	0.50334 (2)	0.48900 (8)	0.01982 (16)	
H5O	0.1448	0.5105	0.4224	0.024*	
O6	0.27845 (11)	0.45380 (2)	0.26950 (7)	0.01601 (14)	
H6O	0.2020	0.4367	0.2481	0.019*	
C1	0.37784 (12)	0.40152 (3)	0.41306 (8)	0.01044 (16)	
H1	0.2638	0.3871	0.3933	0.013*	
C2	0.43530 (12)	0.39073 (3)	0.55006 (9)	0.01108 (16)	
C3	0.29331 (13)	0.40579 (3)	0.64287 (9)	0.01208 (16)	
H3	0.1781	0.3916	0.6249	0.014*	
C4	0.26177 (13)	0.45019 (3)	0.62932 (9)	0.01216 (16)	
H4	0.3735	0.4648	0.6539	0.015*	
C5	0.20777 (13)	0.46107 (3)	0.49359 (9)	0.01271 (17)	
H5	0.0865	0.4499	0.4737	0.015*	
C6	0.34184 (13)	0.44575 (3)	0.39682 (8)	0.01132 (16)	
H6	0.4574	0.4602	0.4102	0.014*	
C7	0.46317 (14)	0.34555 (3)	0.56367 (10)	0.01558 (18)	
H7A	0.5610	0.3374	0.5060	0.019*	
H7B	0.5029	0.3398	0.6524	0.019*	
C8	0.30059 (15)	0.32061 (3)	0.53398 (10)	0.01688 (19)	
C9	0.27011 (18)	0.30651 (4)	0.41014 (12)	0.0247 (2)	
H9	0.3523	0.3128	0.3444	0.030*	
C10	0.1205 (2)	0.28331 (4)	0.38195 (15)	0.0364 (3)	
H10	0.1021	0.2737	0.2974	0.044*	
C11	−0.0017 (2)	0.27418 (4)	0.47645 (18)	0.0414 (4)	
H11	−0.1040	0.2585	0.4569	0.050*	
C12	0.0266 (2)	0.28815 (4)	0.59964 (16)	0.0351 (3)	
H12	−0.0569	0.2821	0.6648	0.042*	
C13	0.17685 (17)	0.31106 (4)	0.62839 (12)	0.0240 (2)	
H13	0.1954	0.3203	0.7133	0.029*	
O1'	−0.02235 (10)	0.60830 (2)	0.78156 (7)	0.01499 (14)	
H1'O	0.0179	0.6122	0.7079	0.018*	
O2'	−0.10251 (10)	0.58343 (2)	1.02760 (7)	0.01449 (14)	
H2'O	−0.1853	0.5942	0.9849	0.017*	
O3'	0.15758 (10)	0.59222 (2)	1.22426 (6)	0.01522 (14)	
H3'O	0.0710	0.5777	1.2446	0.018*	
O4'	0.35488 (10)	0.52508 (2)	1.15761 (7)	0.01644 (15)	
H4'O	0.3179	0.5032	1.1854	0.020*	
O5'	0.30650 (11)	0.49278 (2)	0.90825 (7)	0.01634 (15)	
H5'O	0.4134	0.4869	0.8924	0.020*	
O6'	0.23721 (11)	0.55224 (2)	0.71019 (7)	0.01714 (15)	
H6'O	0.1979	0.5327	0.6691	0.021*	
C1'	0.11963 (12)	0.59546 (3)	0.86443 (8)	0.01071 (16)	
H1'	0.2284	0.6118	0.8465	0.013*	
C2'	0.06277 (12)	0.60309 (3)	1.00248 (8)	0.01054 (16)	
C3'	0.20310 (12)	0.58499 (3)	1.09446 (8)	0.01089 (16)	
H3'	0.3202	0.5983	1.0781	0.013*	
C4'	0.22744 (13)	0.54078 (3)	1.06806 (9)	0.01159 (16)	
H4'	0.1101	0.5269	1.0788	0.014*	
C5'	0.29354 (13)	0.53434 (3)	0.93189 (9)	0.01157 (16)	
H5'	0.4140	0.5469	0.9220	0.014*	
C6'	0.16254 (13)	0.55231 (3)	0.83577 (8)	0.01145 (16)	
H6'	0.0490	0.5366	0.8351	0.014*	
C7'	0.03668 (14)	0.64794 (3)	1.02676 (10)	0.01511 (18)	
H7'A	−0.0651	0.6572	0.9732	0.018*	
H7'B	0.0029	0.6516	1.1173	0.018*	
C8'	0.19624 (14)	0.67385 (3)	0.99995 (10)	0.01606 (18)	
C9'	0.22323 (17)	0.68948 (3)	0.87823 (11)	0.0222 (2)	
H9'	0.1402	0.6836	0.8113	0.027*	
C10'	0.3703 (2)	0.71355 (4)	0.85360 (15)	0.0336 (3)	
H10'	0.3870	0.7241	0.7703	0.040*	
C11'	0.4927 (2)	0.72223 (4)	0.95047 (18)	0.0396 (4)	
H11'	0.5936	0.7385	0.9336	0.048*	
C12'	0.4666 (2)	0.70707 (4)	1.07170 (17)	0.0365 (3)	
H12'	0.5497	0.7130	1.1384	0.044*	
C13'	0.31951 (17)	0.68313 (4)	1.09646 (12)	0.0249 (2)	
H13'	0.3028	0.6730	1.1802	0.030*	
O1W	0.91098 (12)	0.37731 (3)	0.45945 (8)	0.02411 (18)	
H1WA	0.923 (3)	0.3553 (6)	0.4917 (19)	0.036*	
H1WB	0.817 (3)	0.3882 (6)	0.4883 (18)	0.036*	
O2W	0.40295 (12)	0.38183 (3)	0.07198 (8)	0.02373 (18)	
H2WA	0.429 (3)	0.3826 (6)	0.1511 (19)	0.036*	
H2WB	0.429 (3)	0.3596 (6)	0.0440 (18)	0.036*	

### Atomic displacement parameters (Å^2^)

**Table:**

	*U*^11^	*U*^22^	*U*^33^	*U*^12^	*U*^13^	*U*^23^
O1	0.0152 (3)	0.0176 (3)	0.0114 (3)	0.0019 (3)	0.0024 (2)	−0.0026 (2)
O2	0.0117 (3)	0.0222 (4)	0.0106 (3)	−0.0032 (3)	−0.0024 (2)	−0.0004 (2)
O3	0.0227 (4)	0.0257 (4)	0.0089 (3)	0.0054 (3)	0.0015 (3)	0.0026 (3)
O4	0.0161 (3)	0.0180 (4)	0.0138 (3)	0.0009 (3)	0.0039 (2)	−0.0048 (3)
O5	0.0290 (4)	0.0106 (3)	0.0199 (4)	0.0039 (3)	0.0008 (3)	0.0001 (3)
O6	0.0208 (4)	0.0157 (3)	0.0114 (3)	−0.0009 (3)	−0.0036 (3)	0.0036 (2)
C1	0.0105 (4)	0.0115 (4)	0.0093 (4)	0.0011 (3)	−0.0002 (3)	−0.0004 (3)
C2	0.0104 (4)	0.0128 (4)	0.0100 (4)	0.0004 (3)	−0.0009 (3)	0.0004 (3)
C3	0.0127 (4)	0.0143 (4)	0.0093 (4)	0.0009 (3)	0.0007 (3)	0.0009 (3)
C4	0.0120 (4)	0.0128 (4)	0.0117 (4)	0.0000 (3)	0.0020 (3)	−0.0020 (3)
C5	0.0142 (4)	0.0106 (4)	0.0133 (4)	0.0010 (3)	0.0004 (3)	−0.0005 (3)
C6	0.0128 (4)	0.0114 (4)	0.0097 (4)	0.0000 (3)	−0.0005 (3)	0.0007 (3)
C7	0.0160 (4)	0.0140 (4)	0.0168 (4)	0.0033 (3)	−0.0009 (3)	0.0019 (3)
C8	0.0198 (5)	0.0103 (4)	0.0205 (5)	0.0029 (3)	−0.0010 (4)	0.0024 (3)
C9	0.0327 (6)	0.0165 (5)	0.0249 (5)	0.0020 (4)	−0.0045 (4)	−0.0022 (4)
C10	0.0483 (9)	0.0182 (6)	0.0424 (8)	−0.0022 (5)	−0.0191 (6)	−0.0043 (5)
C11	0.0362 (8)	0.0206 (6)	0.0672 (11)	−0.0098 (5)	−0.0168 (7)	0.0085 (6)
C12	0.0281 (6)	0.0232 (6)	0.0541 (9)	−0.0047 (5)	0.0015 (6)	0.0147 (6)
C13	0.0252 (6)	0.0179 (5)	0.0288 (6)	0.0012 (4)	0.0028 (4)	0.0072 (4)
O1'	0.0140 (3)	0.0199 (4)	0.0110 (3)	0.0031 (3)	−0.0019 (2)	0.0028 (2)
O2'	0.0097 (3)	0.0198 (4)	0.0139 (3)	−0.0019 (3)	0.0002 (2)	0.0035 (2)
O3'	0.0168 (3)	0.0196 (4)	0.0093 (3)	−0.0008 (3)	0.0001 (2)	−0.0013 (2)
O4'	0.0172 (3)	0.0157 (3)	0.0163 (3)	0.0016 (3)	−0.0048 (3)	0.0046 (3)
O5'	0.0170 (4)	0.0108 (3)	0.0212 (3)	0.0024 (3)	−0.0001 (3)	−0.0027 (3)
O6'	0.0246 (4)	0.0159 (3)	0.0111 (3)	−0.0030 (3)	0.0038 (3)	−0.0034 (2)
C1'	0.0106 (4)	0.0115 (4)	0.0100 (4)	0.0008 (3)	−0.0007 (3)	0.0004 (3)
C2'	0.0094 (4)	0.0119 (4)	0.0103 (4)	0.0001 (3)	0.0007 (3)	0.0004 (3)
C3'	0.0110 (4)	0.0120 (4)	0.0096 (4)	0.0002 (3)	−0.0006 (3)	−0.0001 (3)
C4'	0.0114 (4)	0.0118 (4)	0.0115 (4)	0.0004 (3)	−0.0016 (3)	0.0014 (3)
C5'	0.0116 (4)	0.0102 (4)	0.0129 (4)	0.0007 (3)	−0.0003 (3)	−0.0004 (3)
C6'	0.0121 (4)	0.0121 (4)	0.0101 (4)	−0.0003 (3)	0.0001 (3)	0.0001 (3)
C7'	0.0161 (4)	0.0129 (4)	0.0163 (4)	0.0039 (3)	0.0016 (3)	−0.0010 (3)
C8'	0.0190 (5)	0.0099 (4)	0.0193 (4)	0.0026 (3)	−0.0009 (3)	−0.0018 (3)
C9'	0.0282 (6)	0.0156 (5)	0.0227 (5)	0.0010 (4)	0.0026 (4)	0.0021 (4)
C10'	0.0416 (8)	0.0177 (5)	0.0417 (7)	−0.0033 (5)	0.0149 (6)	0.0036 (5)
C11'	0.0317 (7)	0.0208 (6)	0.0666 (10)	−0.0102 (5)	0.0100 (7)	−0.0067 (6)
C12'	0.0286 (7)	0.0259 (6)	0.0547 (9)	−0.0043 (5)	−0.0089 (6)	−0.0121 (6)
C13'	0.0264 (6)	0.0202 (5)	0.0278 (6)	0.0008 (4)	−0.0070 (4)	−0.0047 (4)
O1W	0.0194 (4)	0.0309 (5)	0.0221 (4)	0.0063 (3)	0.0047 (3)	0.0091 (3)
O2W	0.0206 (4)	0.0337 (5)	0.0168 (4)	0.0071 (3)	−0.0031 (3)	−0.0046 (3)

### Geometric parameters (Å, °)

**Table:**

O1—C1	1.4259 (11)	O2'—C2'	1.4275 (11)
O1—H1O	0.8400	O2'—H2'O	0.8400
O2—C2	1.4299 (12)	O3'—C3'	1.4233 (11)
O2—H2O	0.8400	O3'—H3'O	0.8400
O3—C3	1.4222 (11)	O4'—C4'	1.4291 (11)
O3—H3O	0.8400	O4'—H4'O	0.8400
O4—C4	1.4358 (12)	O5'—C5'	1.4284 (12)
O4—H4O	0.8400	O5'—H5'O	0.8400
O5—C5	1.4293 (12)	O6'—C6'	1.4315 (11)
O5—H5O	0.8400	O6'—H6'O	0.8400
O6—C6	1.4336 (11)	C1'—C6'	1.5223 (13)
O6—H6O	0.8400	C1'—C2'	1.5303 (12)
C1—C6	1.5268 (13)	C1'—H1'	1.0000
C1—C2	1.5343 (12)	C2'—C3'	1.5403 (13)
C1—H1	1.0000	C2'—C7'	1.5481 (14)
C2—C3	1.5312 (13)	C3'—C4'	1.5295 (13)
C2—C7	1.5460 (14)	C3'—H3'	1.0000
C3—C4	1.5240 (14)	C4'—C5'	1.5269 (13)
C3—H3	1.0000	C4'—H4'	1.0000
C4—C5	1.5159 (13)	C5'—C6'	1.5206 (13)
C4—H4	1.0000	C5'—H5'	1.0000
C5—C6	1.5209 (13)	C6'—H6'	1.0000
C5—H5	1.0000	C7'—C8'	1.5064 (15)
C6—H6	1.0000	C7'—H7'A	0.9900
C7—C8	1.5064 (15)	C7'—H7'B	0.9900
C7—H7A	0.9900	C8'—C13'	1.3935 (16)
C7—H7B	0.9900	C8'—C9'	1.3941 (15)
C8—C9	1.3957 (16)	C9'—C10'	1.3921 (18)
C8—C13	1.3964 (16)	C9'—H9'	0.9500
C9—C10	1.3925 (19)	C10'—C11'	1.387 (2)
C9—H9	0.9500	C10'—H10'	0.9500
C10—C11	1.386 (3)	C11'—C12'	1.382 (3)
C10—H10	0.9500	C11'—H11'	0.9500
C11—C12	1.385 (3)	C12'—C13'	1.390 (2)
C11—H11	0.9500	C12'—H12'	0.9500
C12—C13	1.3921 (19)	C13'—H13'	0.9500
C12—H12	0.9500	O1W—H1WA	0.82 (2)
C13—H13	0.9500	O1W—H1WB	0.85 (2)
O1'—C1'	1.4286 (11)	O2W—H2WA	0.85 (2)
O1'—H1'O	0.8400	O2W—H2WB	0.83 (2)
			
C1—O1—H1O	109.5	C2'—O2'—H2'O	109.5
C2—O2—H2O	109.5	C3'—O3'—H3'O	109.5
C3—O3—H3O	109.5	C4'—O4'—H4'O	109.5
C4—O4—H4O	109.5	C5'—O5'—H5'O	109.5
C5—O5—H5O	109.5	C6'—O6'—H6'O	109.5
C6—O6—H6O	109.5	O1'—C1'—C6'	109.10 (7)
O1—C1—C6	110.28 (7)	O1'—C1'—C2'	108.02 (7)
O1—C1—C2	110.50 (7)	C6'—C1'—C2'	114.06 (8)
C6—C1—C2	112.54 (7)	O1'—C1'—H1'	108.5
O1—C1—H1	107.8	C6'—C1'—H1'	108.5
C6—C1—H1	107.8	C2'—C1'—H1'	108.5
C2—C1—H1	107.8	O2'—C2'—C1'	110.12 (7)
O2—C2—C3	111.39 (8)	O2'—C2'—C3'	106.47 (7)
O2—C2—C1	105.48 (7)	C1'—C2'—C3'	109.16 (7)
C3—C2—C1	108.91 (8)	O2'—C2'—C7'	108.32 (8)
O2—C2—C7	108.77 (8)	C1'—C2'—C7'	110.85 (8)
C3—C2—C7	111.27 (8)	C3'—C2'—C7'	111.82 (8)
C1—C2—C7	110.87 (8)	O3'—C3'—C4'	111.68 (8)
O3—C3—C4	111.05 (8)	O3'—C3'—C2'	111.06 (8)
O3—C3—C2	107.39 (8)	C4'—C3'—C2'	110.85 (7)
C4—C3—C2	112.03 (8)	O3'—C3'—H3'	107.7
O3—C3—H3	108.8	C4'—C3'—H3'	107.7
C4—C3—H3	108.8	C2'—C3'—H3'	107.7
C2—C3—H3	108.8	O4'—C4'—C5'	109.79 (8)
O4—C4—C5	107.50 (8)	O4'—C4'—C3'	108.87 (8)
O4—C4—C3	109.47 (8)	C5'—C4'—C3'	110.32 (7)
C5—C4—C3	111.39 (8)	O4'—C4'—H4'	109.3
O4—C4—H4	109.5	C5'—C4'—H4'	109.3
C5—C4—H4	109.5	C3'—C4'—H4'	109.3
C3—C4—H4	109.5	O5'—C5'—C6'	108.79 (8)
O5—C5—C4	106.49 (8)	O5'—C5'—C4'	108.92 (8)
O5—C5—C6	110.13 (8)	C6'—C5'—C4'	110.32 (8)
C4—C5—C6	111.71 (8)	O5'—C5'—H5'	109.6
O5—C5—H5	109.5	C6'—C5'—H5'	109.6
C4—C5—H5	109.5	C4'—C5'—H5'	109.6
C6—C5—H5	109.5	O6'—C6'—C5'	110.57 (8)
O6—C6—C5	109.88 (8)	O6'—C6'—C1'	105.48 (7)
O6—C6—C1	110.16 (7)	C5'—C6'—C1'	112.77 (8)
C5—C6—C1	112.02 (8)	O6'—C6'—H6'	109.3
O6—C6—H6	108.2	C5'—C6'—H6'	109.3
C5—C6—H6	108.2	C1'—C6'—H6'	109.3
C1—C6—H6	108.2	C8'—C7'—C2'	115.90 (8)
C8—C7—C2	115.18 (8)	C8'—C7'—H7'A	108.3
C8—C7—H7A	108.5	C2'—C7'—H7'A	108.3
C2—C7—H7A	108.5	C8'—C7'—H7'B	108.3
C8—C7—H7B	108.5	C2'—C7'—H7'B	108.3
C2—C7—H7B	108.5	H7'A—C7'—H7'B	107.4
H7A—C7—H7B	107.5	C13'—C8'—C9'	118.36 (11)
C9—C8—C13	118.31 (11)	C13'—C8'—C7'	120.85 (10)
C9—C8—C7	120.31 (10)	C9'—C8'—C7'	120.79 (10)
C13—C8—C7	121.38 (10)	C10'—C9'—C8'	120.78 (12)
C10—C9—C8	120.70 (13)	C10'—C9'—H9'	119.6
C10—C9—H9	119.7	C8'—C9'—H9'	119.6
C8—C9—H9	119.7	C11'—C10'—C9'	120.16 (13)
C11—C10—C9	120.40 (14)	C11'—C10'—H10'	119.9
C11—C10—H10	119.8	C9'—C10'—H10'	119.9
C9—C10—H10	119.8	C12'—C11'—C10'	119.53 (13)
C12—C11—C10	119.47 (13)	C12'—C11'—H11'	120.2
C12—C11—H11	120.3	C10'—C11'—H11'	120.2
C10—C11—H11	120.3	C11'—C12'—C13'	120.35 (13)
C11—C12—C13	120.26 (14)	C11'—C12'—H12'	119.8
C11—C12—H12	119.9	C13'—C12'—H12'	119.8
C13—C12—H12	119.9	C12'—C13'—C8'	120.83 (13)
C12—C13—C8	120.86 (13)	C12'—C13'—H13'	119.6
C12—C13—H13	119.6	C8'—C13'—H13'	119.6
C8—C13—H13	119.6	H1WA—O1W—H1WB	109.3 (18)
C1'—O1'—H1'O	109.5	H2WA—O2W—H2WB	108.5 (18)
			
O1—C1—C2—O2	59.12 (9)	O1'—C1'—C2'—O2'	56.69 (10)
C6—C1—C2—O2	−64.64 (10)	C6'—C1'—C2'—O2'	−64.78 (10)
O1—C1—C2—C3	178.79 (8)	O1'—C1'—C2'—C3'	173.24 (7)
C6—C1—C2—C3	55.03 (10)	C6'—C1'—C2'—C3'	51.76 (10)
O1—C1—C2—C7	−58.46 (10)	O1'—C1'—C2'—C7'	−63.16 (10)
C6—C1—C2—C7	177.78 (8)	C6'—C1'—C2'—C7'	175.36 (8)
O2—C2—C3—O3	−62.82 (10)	O2'—C2'—C3'—O3'	−62.52 (10)
C1—C2—C3—O3	−178.74 (8)	C1'—C2'—C3'—O3'	178.63 (7)
C7—C2—C3—O3	58.74 (10)	C7'—C2'—C3'—O3'	55.61 (10)
O2—C2—C3—C4	59.37 (10)	O2'—C2'—C3'—C4'	62.25 (9)
C1—C2—C3—C4	−56.55 (10)	C1'—C2'—C3'—C4'	−56.59 (10)
C7—C2—C3—C4	−179.06 (8)	C7'—C2'—C3'—C4'	−179.61 (8)
O3—C3—C4—O4	−64.21 (10)	O3'—C3'—C4'—O4'	−54.04 (10)
C2—C3—C4—O4	175.71 (7)	C2'—C3'—C4'—O4'	−178.46 (7)
O3—C3—C4—C5	177.05 (8)	O3'—C3'—C4'—C5'	−174.57 (7)
C2—C3—C4—C5	56.96 (10)	C2'—C3'—C4'—C5'	61.00 (10)
O4—C4—C5—O5	65.91 (10)	O4'—C4'—C5'—O5'	62.81 (10)
C3—C4—C5—O5	−174.17 (8)	C3'—C4'—C5'—O5'	−177.20 (7)
O4—C4—C5—C6	−173.82 (8)	O4'—C4'—C5'—C6'	−177.84 (8)
C3—C4—C5—C6	−53.90 (11)	C3'—C4'—C5'—C6'	−57.86 (10)
O5—C5—C6—O6	−66.62 (10)	O5'—C5'—C6'—O6'	−70.13 (10)
C4—C5—C6—O6	175.27 (8)	C4'—C5'—C6'—O6'	170.45 (8)
O5—C5—C6—C1	170.59 (8)	O5'—C5'—C6'—C1'	172.04 (8)
C4—C5—C6—C1	52.47 (11)	C4'—C5'—C6'—C1'	52.62 (10)
O1—C1—C6—O6	59.58 (10)	O1'—C1'—C6'—O6'	67.44 (9)
C2—C1—C6—O6	−176.54 (7)	C2'—C1'—C6'—O6'	−171.68 (8)
O1—C1—C6—C5	−177.79 (7)	O1'—C1'—C6'—C5'	−171.78 (7)
C2—C1—C6—C5	−53.91 (10)	C2'—C1'—C6'—C5'	−50.90 (11)
O2—C2—C7—C8	−175.19 (8)	O2'—C2'—C7'—C8'	−177.69 (8)
C3—C2—C7—C8	61.74 (11)	C1'—C2'—C7'—C8'	−56.77 (11)
C1—C2—C7—C8	−59.64 (11)	C3'—C2'—C7'—C8'	65.30 (11)
C2—C7—C8—C9	91.19 (12)	C2'—C7'—C8'—C13'	−92.33 (12)
C2—C7—C8—C13	−88.68 (12)	C2'—C7'—C8'—C9'	88.33 (12)
C13—C8—C9—C10	−0.38 (17)	C13'—C8'—C9'—C10'	0.40 (17)
C7—C8—C9—C10	179.75 (11)	C7'—C8'—C9'—C10'	179.76 (11)
C8—C9—C10—C11	0.7 (2)	C8'—C9'—C10'—C11'	0.1 (2)
C9—C10—C11—C12	−0.4 (2)	C9'—C10'—C11'—C12'	−0.5 (2)
C10—C11—C12—C13	−0.2 (2)	C10'—C11'—C12'—C13'	0.3 (2)
C11—C12—C13—C8	0.5 (2)	C11'—C12'—C13'—C8'	0.2 (2)
C9—C8—C13—C12	−0.21 (17)	C9'—C8'—C13'—C12'	−0.58 (18)
C7—C8—C13—C12	179.66 (11)	C7'—C8'—C13'—C12'	−179.94 (11)

### Hydrogen-bond geometry (Å, °)

**Table:**

*D*—H···*A*	*D*—H	H···*A*	*D*···*A*	*D*—H···*A*
O1'—H1'O···O1W^i^	0.84	1.87	2.7052 (11)	175
O1—H1O···O6'^i^	0.84	1.94	2.7768 (10)	171
O2'—H2'O···O2W^ii^	0.84	1.90	2.7267 (12)	166
O2—H2O···O3'^iii^	0.84	2.04	2.7755 (10)	145
O3'—H3'O···O4^iv^	0.84	2.01	2.8420 (10)	174
O3—H3O···O2'^iv^	0.84	2.09	2.9290 (11)	172
O4'—H4'O···O6^v^	0.84	1.91	2.7389 (10)	168
O4—H4O···O5'	0.84	1.87	2.6858 (10)	165
O5'—H5'O···O4'^iii^	0.84	1.86	2.6943 (11)	175
O5—H5O···O4^ii^	0.84	2.57	3.3617 (11)	157
O6'—H6'O···O5	0.84	2.13	2.8523 (11)	144
O6—H6O···O1'^ii^	0.84	2.05	2.8831 (10)	173
O1W—H1WB···O2	0.85 (2)	1.96 (2)	2.8108 (12)	174.2 (19)
O2W—H2WA···O1	0.85 (2)	1.91 (2)	2.7521 (11)	173.3 (18)
O1W—H1WA···Cg1^vi^	0.82 (2)	2.59 (2)	3.2647 (11)	140.9 (19)
O2W—H2WB···Cg2^i^	0.83 (2)	2.59 (2)	3.3335 (11)	149.9 (19)

Symmetry codes: (i) −*x*+1, −*y*+1, −*z*+1; (ii) −*x*, −*y*+1, −*z*+1; (iii) −*x*+1, −*y*+1, −*z*+2; (iv) −*x*, −*y*+1, −*z*+2; (v) *x*, *y*, *z*+1; (vi) *x*+1, *y*, *z*.
